# Barriers against implementing assertive community treatment teams in Spain: a qualitative exploration of the staff, users and general population experiences

**DOI:** 10.1192/j.eurpsy.2025.2173

**Published:** 2025-08-26

**Authors:** J. J. M. Jambrina, A. D. Rivas, J. M. Vela, C. O. Roldán

**Affiliations:** 1 Mental Health, Hospital Universitario San Agustín, Avilés; 2 Mental Health, Hospital Universitario Arquitecto Marcide, Ferrol; 3 Mental Health, Hospital Universitario Central de Asturias, Oviedo, Spain

## Abstract

**Introduction:**

Three years after having been approved the Spanish Strategy for Mental Health, the creation of Assertive Community treatment teams is getting slower.

**Objectives:**

The current study seeks to investigate the reasons of and barriers to implementation of this approach. Findings will aim at serving to inform mental health policy about legislative changes.

**Methods:**

Qualitative expert interviews and focus groups were led with team members and users, families and general population from the cities where four of this teams have been developing theirs activities, at least during 15 years A thematic analysis was developed.

**Results:**

Four main topics were identified: 1. Stigma: when economical situation is not good enough, people with psychiatric severe illnesses are not the first in the queue 2. Fear and distrust about breaking the CMHCenters policies; 2. Team development: a hard task more than a challenge; 3. Regardless of profession, finding well trained staff is difficult;

**Conclusions:**

Further research is needed to convince politicians and health authorities. Randomized controlled trials examining the efficacy and efficiency of ACT teams are needed

**Disclosure of Interest:**

None Declared

